# Repeated fMRI in measuring the activation of the amygdala without habituation when viewing faces displaying negative emotions

**DOI:** 10.1371/journal.pone.0198244

**Published:** 2018-06-04

**Authors:** Jennifer Spohrs, Julia E. Bosch, Lisa Dommes, Petra Beschoner, Julia C. Stingl, Franziska Geiser, Katharina Schneider, Jörg Breitfeld, Roberto Viviani

**Affiliations:** 1 Department of Psychiatry and Psychotherapy III, University of Ulm, Ulm, Germany; 2 Psychosomatics and Psychotherapy Clinic, University of Ulm, Ulm, Germany; 3 Federal Institute for Drugs and Medical Devices and Centre for Translational Medicine, University of Bonn Medical School, Bonn, Germany; 4 Clinic for Psychosomatic Medicine and Psychotherapy, University of Bonn, Bonn, Germany; 5 Institute of Psychology, University of Innsbruck, Innsbruck, Austria; Harvard Medical School, UNITED STATES

## Abstract

Functional imaging studies of affective disorders have demonstrated abnormal activity in the amygdala in response to emotionally salient stimuli. Since in other studies this response has been shown to habituate during the scanning session, it is not clear if it may be of use in monitoring disease progression or remission, or in monitoring the effects of therapy, as habituation may confound normalisation of response. We investigated here amygdala activation in healthy participants exposed to displays of emotional facial expressions in a sample of N = 31 individuals assessed twice in an interval of three weeks. At this interval no habituation could be detected, suggesting the validity of this imaging assay in repeated assessments of amygdalar reactivity. However, the fusiform gyrus and the inferior frontal lobes showed decreases in activations that may be related to the role of these areas in encoding visual and emotional aspects of the stimuli.

## Introduction

The amygdala, a bilateral structure in the anteromedial temporal lobe, plays a critical role in the identification of emotionally salient events and in fear conditioning [1–3]. Many cues and stimuli have been used in studies with primates and humans to stimulate the amygdala. It has been shown that faces containing emotional cues reliably activate the amygdala in functional imaging (fMRI) studies [1, 4–6]. Typically, facial expressions depicting aversive emotions, such as fear, anger, or disgust show a stronger increase in the activation of the amygdala than neutral or positive facial expressions [4, 5].

Functional MRI studies have also provided evidence for the involvement of the amygdala in affective disorders [7, 8]. These findings motivated several fMRI studies where activation of the amygdala by facial expressions was used as an in vivo assay to monitor the effectiveness and progress of therapy of affective disorders [9–11]. However, after repeated presentations the response of the amygdala to salient stimuli, not followed by significant consequences, habituates [12]. Habituation of the amygdala has also been demonstrated in fMRI studies, as when repeatedly exposing participants to emotionally arousing stimuli such as negatively valenced images [13] or facial expressions [14–19]. Subsequent studies reported that this habituation may be attenuated in affective disorders [20, 21]. Therefore, the evolution over time of the fMRI signal from the amygdala seems to be a critical element to discriminate between habituation due to repeated presentation of the assay and changes in primary amygdala reactivity due to possible changes in affective processing after therapy.

While these studies documented habituation in the amygdala within the scanning session, little is known about the extent of habituation in repeated presentations over weeks, as it would be needed to monitor the course of therapy. To examine whether habituation effects, potentially biasing the analysis, occur or not, we scanned a group of healthy subjects with the same fMRI assay of emotional face expressions twice over an interval of three weeks (the short end of the interval range in which the effectiveness of therapy of depression is typically assessed). The analysis took place in the control group of healthy individuals within a larger study in depressed patients analysing if non-invasive imaging methods are suitable for monitoring disease outcome.

## Methods

### Functional imaging paradigm

The paradigm used in the present study is based on the Karolinska Directed Emotional Faces picture set [22]. For the study at hand 192 pictures were selected and divided into 6 blocks. In each block 32 pictures of faces expressing sadness, anger, or disgust were presented to the participant in a pseudorandomised order. The faces in this image set display a generic northern European look, similar to the general appearance of individuals in the population in which the sample was recruited. As an implicit baseline, in the second part of each block a sequence of 32 geometric figures, such as triangles, squares, circles and rectangles followed the presentation of the pictures. The duration of each block was 32 seconds and the pictures of faces and geometric images were presented for 500 msec. In total the paradigm lasted approximately 3.30 mins. The participants were able to look at the images by means of a mirror integrated into their headgear, which allowed them to look at a screen projected behind the scanner. To program the trials the standard software Presentation 14 (Neurobehavioral Systems Inc., Albany, CA) was used.

### Recruitment and data acquisition

The study was conducted at two locations: the Federal Institute for Drugs and Medical Devices in Bonn and the University Clinic for Psychiatry and Psychotherapy III, Ulm, Germany. This study was conducted in conformance with the guidelines of the Declaration of Helsinki and was approved by the Ethical committee of the University of Bonn and the Ethical committee of the University of Ulm as part of a larger study on imaging as monitoring assay in patients suffering a Major Depressive Disorder and healthy patients. To analyse the potential habituation effects that might occur when subjects are presented to the same paradigm after a time-lag, the present report focusses exclusively on the healthy controls of the study. The data acquisition took place at two different points in time with an average time-lag of three weeks (in a range from 18 to 24 days), which corresponds to the time-lag (10–20 days) for the onset of the effects of common antidepressant drug therapies.

Healthy volunteers of European origin were recruited via placards in local facilities and admitted to the study if no exclusion criteria were met (current alcohol/drug addiction, anorexia, or current affective psychological disorders, pregnancy/lactation, severe acute or chronic diseases, long-term medication intake, metal implants, large tattoos or tattoos near the head). Additionally, all participants had to sign a written informed consent after a briefing on the study. In total 38 healthy individuals participated in the control arm of the study, but seven had to be excluded from the analysis for not attending the second MRI session, leaving 31 participants in the sample (men = 12, women = 19). Data were collected with a 3T Siemens Prisma (in the Psychiatry and Psychotherapy Clinic of the University, Ulm) and a 3T Siemens Skyra scanner (located on the premises of the German Center for Neurodegenerative Diseases, DZNE, Bonn) equipped with 64-channels head coils with a T2*-sensitive echo planar imaging sequence (TR/TE: 2460/30msec), flip angle 82°, FOV 24 cm, 64x64 pixels of 3x3mm in 39 2.5 mm transversal slices (in ascending acquisition order) with a gap of .5mm, giving an isotropic pixel size of 3mm. To improve the detection of the MRI signal from the amygdala and to reduce the impact of susceptibility artefacts, a variable echo was used as described in ref. [23]. Echo time was gradually shortened by 8msec from slice 24 to slice 14, giving a TE of 22msec in the first 14 slices acquired in the bottom of the volume.

### Data analysis

**Fig 1:**
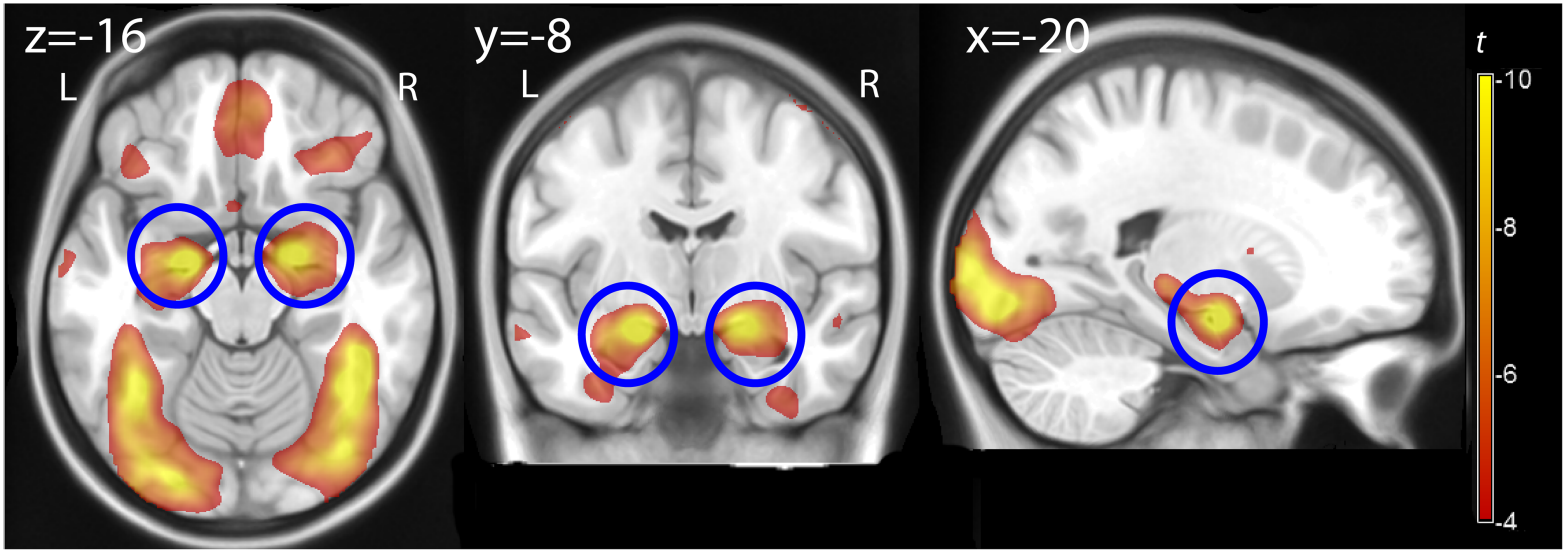
Effect of faces. Statistical parametric map of the main effect of the contrast images faces vs. control, overlaid on a template brain, assessing the average effect of passive exposure to faces at both measurements. Color represents *t* values computed voxelwise, thresholded for illustration at *p* = .05, FWE-corrected. The blue circles indicate the location of the amygdala. L, R: left, right.

**Fig 2:**
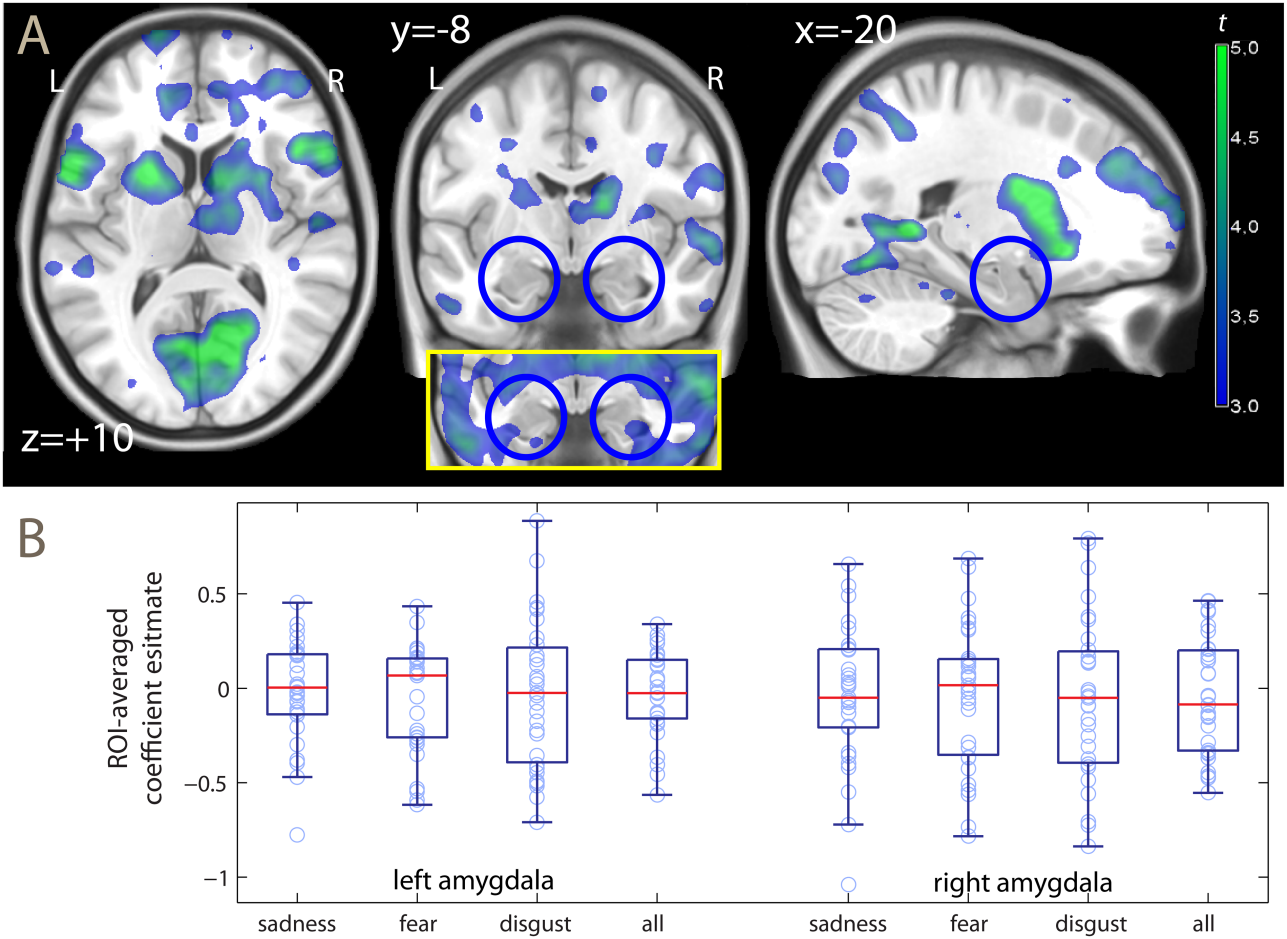
Habituation effect. A: Statistical parametric maps of the reduction in activity from the first and second measurement, overlaid on a template brain. Color represents *t* values computed voxelwise, thresholded for illustration at *p* = .001, uncorrected. The inset shows the amygdala region at the uncorrected threshold *p* = .05. The blue circles indicate the location of the amygdala. L, R: left, right. B: box-plots of the estimated habituation effect in the ROI-averaged signal (negative values represent decrements at the second measurement). Data available in the S1 Supplementary Information.

To analyse the data, the software SPM12 (Wellcome Trust Centre for Neuroimaging, http://www.fil.ion.ucl.ac.uk/spm/) running on MATLAB (TheMathWorks) was used. After realignment, normalisation, and smoothing (FWHM 8mm) data were regressed at the first level on a box-car function convolved with a canonical haemodynamic function and the realignment parameters as confounding covariates. Separate regressors and intercept terms modelled the two sessions (for results reported in Figs 1 and 2 and Table 1). An ancillary model, used in the S1 Supplementary Information and in Fig 2 to visualize effects in each emotional expression, consisted of separate regressors for each of the emotions used in the study. The coefficients of these regressions, representing the effect of face presentation relative to the implicit baseline of the geometric figures and the interaction with sessions, were brought to the second level to account for the random effects of subjects. At the second level, significance tests were computed with random field theory correction at the cluster level, with clusters defined a priori by the threshold p < .001, uncorrected. Regions of interest analyses on the amygdala were conducted using an anatomical mask [24]. Overlays were produced with the freely available software MRIcron (http://people.cas.sc.edu/rorden/mricron/index.html). Designations of cortical areas in tables were obtained from the ‘aal’ atlas [25] provided with this software package.

**Table 1:** Decreases in activation from first to second measurement.

Cluster	Location	MNI coord.	*k*	*p* clust.	*t*	*p* peak
1	R Calcarine/Lingual (BA17/18)	10–64 10	6327	< .001	-6.76	.01
	L Calcarine/Lingual (BA17/18)	-6–68 10			-4.26	.22
	L Lingual/Fusiform (BA18/19)	-22–68–12			-4.15	.30
2	L Putamen/Caudate	-22 8 1	5294	< .001	-6.06	.03
	R Putamen/Caudate	22 24 4			-5.69	.07
3	R Supp. Motor (BA6)	4 12 66	1332	< .001	-4.82	.38
4	L Frontal Inferior (BA6/48)	-56 8 10	726	.001	-5.64	.08
	L Frontal Operc. (BA45/47/48)	-42 25 4			-5.40	.13
5	R Frontal Inferior (BA45/48)	46 20 10	405	.021	-5.03	.27
6	L Mid./Inf. Temporal (BA20/21)	-56–36–12	511	.008	-4.85	.36
	L Mid. Temporal (BA20/21)	-50–16–10			-3.58	.88
7	R Sup./Mid. Temporal (BA21)	64–2–8	363	.031	-3.85	.61

MNI coord: Montreal Neurological Institute coordinated (in mm); *k*: cluster extent (in voxel of isotropic size 2 mm); *p* clust.: significance level, cluster level correction for the whole volume; *t*: Student’s *t*; *p* peak: significance level, peak-level correction for the whole volume; BA: Brodmann area; Inf., Mid., Sup: inferior, middle, superior; Operc.: operculum; Supp: supplementary; R, L: right, left

## Results

As expected, the contrast between the images of the faces expressing aversive emotions and the baseline given by the geometric figures gave significant bilateral activations of the amygdala (right amygdala: Montreal Neurological Institute coordinates x, y, z: -20, -8, -16, *t* = 14.33, peak-level corrected *p* < .001, left amygdala: x, y, z: 20, -6, -14, *t* = 11.89, peak-level corrected *p* < .001) (Fig 1). Additionally, activations were observed in the inferior temporal lobe/gyrus fusiformis (x, y, z: 44, -44, -20, *t* = 17.29; -38, -54, -18, *t* = 12.73), the visual cortex (x, y, z: -18, -96, 0, *t* = 16.99; 20, -92, -4, *t* = 7.73), the right precentral, in the right inferior frontal gyrus (x, y, z: 50, 8, 54, *t* = 9.17; x, y, z: 46, 26, 20, *t* = 9.76, all peak-level corrected *p* < .001) and in the medial orbitofrontal cortex (x, y, z: 2, 46, -20, *t* = 7.69, *p* = .002, peak-level corrected).

The main contrast of interest tested the decrease of the activation of the amygdala relative to the baseline from the first to the second measurement (interaction face presentation × sessions; see and Fig 2). Against the hypothesis that a habituation may occur and result in a decrease of the activation of the amygdala, this effect was not significant. When averaging the signal from the region of interest (ROI) of the amygdala (panel B of Fig 2), no significant change was detected on either side (left, *t* = -0.88; right, *t* = -0.82, not significant) or when the data were pooled together (*t* = -0.88, not significant; see S1 Supplementary Information for the data and test statistics in the separate emotions). Likewise, no significance change was detected when testing for the presence of habituation voxel by voxel, using a ROI-based correction for multiple testing. This null finding did not change after adding nuisance covariates to the analysis (gender, age, and acquisition site; right amygdala, x, y, z: 32, -2, -30, *t* = -2.38, not significant; left amygdala, x, y, z: -20, -2, -30, *t* = -2.40, not significant) or when we tested the clusters in this ROI at a more lenient cluster-defining threshold (*p* < .05 uncorrected). Indeed, inspection of the uncorrected map (shown in the inset of Fig 2) reveals that the decrements of activation in the amygdala detected at uncorrected levels arose from encroachments from the adjacent parahippocampal cortex.

We then tested the same contrast in the whole brain, applying a correction for the whole volume (Table 1). Here, significant activation decrements after three weeks were detected in several regions, including the pallidum/caudate, the fusiform gyrus, the inferior and medial temporal gyri in their posterior portion straddling over the occipital cortex and the inferior frontal gyrus/frontal operculum. A comparison of these reductions with the task activations revealed an overlap in the inferior frontal gyrus/frontal operculum.

There was no significant signal increase after three weeks.

## Discussion

The amygdala is a key component of neural circuits that are highly preserved across species and are tasked with the recognition and expression of negative motivational states [26, 27]. As an fMRI endophenotype of affective disorder, the importance of the activation of the amygdala under passive exposure to faces with emotional expressions may be related to individual differences in reactivity and arousal to negatively valenced stimuli [1]. The aim of the present study was to investigate whether this fMRI paradigm can be used in repeated measurements or if changes in signal intensity in the amygdala are observed. In contrast to literature reports made when repeating exposure to faces or similarly arousing stimuli within the same scanning session, there was no significant decrease in the activation of the amygdala after three weeks.

A limitation of the present study was the size of the sample (n = 31). However, the studies available in the literature have been able to demonstrate habituation to facial stimuli in much smaller samples (at most n = 10), suggesting that any habituation in the amygdala, if present at all, must be much smaller than in the within-session setting. Furthermore, the present study provides no information on neutral faces, which have also been shown to habituate at short intervals [14].

It has long been noted that emotional stimuli have the capacity to focus attention and are preferentially processed [28]. There is considerable evidence that the amygdala plays a key role in this process through its subcortical inputs and reciprocal connections to the visual cortex [2, 29, 30]. The disappearance of habituation after days or weeks is consistent with this function, as it would make no evolutionary sense for a mechanism detecting emotional salience to stop working after a first exposure.

A perhaps surprising finding of the present study was the detection of decreases of cortical activations after three weeks. In the neuroimaging litarature, decreases of the signal at repeated presentation of stimuli is a robust finding known as repetition suppression, and is thought to reflect an adapation of neural coding related to implicit memory of stimuli [31, 32]. The location of the effect is selective for the features that area repeated [33], and is consistent with the decreases observed here. The fusiform gyrus hosts the face recognition area [6]. The inferior frontal gyrus and the frontal operculum have been involved in processing emotional information [34]. Repetition suppression has previously been reported for faces and facial expressions [35] (see also [13, 36]), and is distinct from and does not depend on explicit recollection [32]. This finding suggests that implicit memory of stimuli, such as conceptual or sensory priming or familiarity, did have an effect on cortical activations detectable with fMRI after three weeks, but one that involved high-level cortical representations rather than the reactivity detected in the amygdala. Repetition suppression in neuroimaging data has been detected after three days [37], but its persistence over weeks has not been previously reported. However, behavioural studies have shown that sensory priming is still present after at least 48 weeks [38].

## Supporting information

S1 Supplementary Information (DOC)
