# Supplementary material for: Repeated fMRI in measuring the activation of the amygdala without habituation when viewing faces displaying negative emotions
Source: PLoS One. 2018 Jun 4;13(6):e0198244. doi: 10.1371/journal.pone.0198244 (PMC5986117; doi:10.1371/journal.pone.0198244)
Supplement: S1 Supplementary Information — (DOC) [file pone.0198244.s001.doc]

### Supplementary Information

The table contains the full ROI-averaged dataset of the estimated habituation effect (interaction session x effect of faces relative to the implicit baseline) obtained at the first level. Negative values indicate reduction of the faces effect in the second session, and are consistent with a habituation effect. The estimates were obtained with two models: one in which the effects of the individual emotions were modelled as separate regressors, and one in which all emotions were pooled together (as in the main text). The data are displayed graphically in Figure 2B of the main text.

| Subject | sadness L | fear L | disgust L | all L | sadness R | fear R | disgust R | all R |
| --- | --- | --- | --- | --- | --- | --- | --- | --- |
| 1 | -0.778 | -0.132 | -0.517 | -0.457 | -1.039 | -0.001 | -0.006 | -0.340 |
| 2 | 0.193 | -0.242 | 0.023 | -0.020 | 0.227 | 0.060 | -0.041 | 0.080 |
| 3 | -0.396 | -0.617 | -0.446 | -0.564 | -0.359 | -0.540 | -0.162 | -0.475 |
| 4 | 0.181 | 0.163 | 0.124 | 0.153 | 0.351 | 0.157 | 0.133 | 0.208 |
| 5 | 0.179 | -0.223 | -0.424 | -0.158 | 0.032 | -0.313 | -0.706 | -0.328 |
| 6 | -0.110 | -0.261 | -0.578 | -0.189 | -0.339 | -0.365 | -0.726 | -0.367 |
| 7 | -0.072 | -0.224 | -0.122 | -0.134 | -0.201 | -0.020 | -0.051 | -0.090 |
| 8 | 0.307 | 0.107 | -0.024 | 0.152 | 0.492 | 0.116 | 0.204 | 0.303 |
| 9 | 0.123 | 0.155 | 0.266 | 0.176 | 0.053 | 0.016 | 0.169 | 0.076 |
| 10 | -0.067 | -0.545 | -0.492 | -0.365 | -0.133 | -0.735 | -0.559 | -0.466 |
| 11 | -0.142 | 0.085 | -0.404 | -0.180 | -0.208 | 0.075 | -0.403 | -0.199 |
| 12 | -0.470 | -0.044 | -0.174 | -0.234 | -0.549 | -0.054 | -0.374 | -0.331 |
| 13 | 0.004 | 0.068 | 0.061 | 0.042 | -0.066 | -0.288 | -0.089 | -0.149 |
| 14 | -0.025 | 0.188 | 0.044 | 0.125 | -0.102 | 0.352 | 0.144 | 0.210 |
| 15 | 0.453 | 0.161 | 0.428 | 0.340 | 0.658 | 0.372 | 0.380 | 0.462 |
| 16 | 0.221 | -0.294 | 0.124 | 0.008 | 0.319 | -0.510 | -0.022 | -0.086 |
| 17 | 0.173 | 0.213 | -0.355 | -0.026 | 0.018 | -0.077 | -0.308 | -0.153 |
| 18 | 0.080 | 0.203 | -0.502 | -0.051 | 0.319 | 0.310 | -0.255 | 0.146 |
| 19 | -0.038 | -0.533 | 0.230 | -0.129 | -0.076 | -0.427 | 0.139 | -0.132 |
| 20 | 0.021 | -0.590 | -0.709 | -0.404 | -0.205 | -0.367 | -0.838 | -0.445 |
| 21 | 0.268 | 0.348 | -0.079 | 0.184 | 0.543 | 0.687 | -0.093 | 0.407 |
| 22 | 0.181 | -0.351 | 0.887 | 0.148 | -0.419 | -0.562 | 0.260 | -0.346 |
| 23 | -0.382 | 0.142 | -0.223 | -0.160 | -0.399 | -0.109 | -0.401 | -0.288 |
| 24 | -0.143 | 0.154 | 0.174 | 0.055 | 0.011 | 0.054 | -0.194 | -0.045 |
| 25 | 0.011 | 0.139 | 0.676 | 0.241 | 0.205 | 0.320 | 0.793 | 0.414 |
| 26 | -0.299 | -0.259 | -0.039 | -0.134 | -0.722 | -0.784 | -0.415 | -0.554 |
| 27 | 0.005 | -0.279 | 0.151 | -0.051 | 0.070 | 0.058 | 0.365 | 0.162 |
| 28 | -0.207 | 0.066 | -0.243 | -0.127 | -0.050 | 0.477 | -0.486 | -0.039 |
| 29 | 0.341 | 0.079 | 0.420 | 0.284 | 0.209 | 0.135 | 0.638 | 0.331 |
| 30 | -0.019 | 0.434 | 0.458 | 0.257 | 0.100 | 0.643 | 0.771 | 0.464 |
| 31 | -0.127 | 0.159 | 0.367 | 0.119 | -0.066 | 0.147 | 0.484 | 0.180 |
| median | 0.004 | 0.068 | -0.024 | -0.026 | -0.050 | 0.016 | -0.051 | -0.086 |
| mean | -0.017 | -0.056 | -0.029 | -0.035 | -0.043 | -0.038 | -0.053 | -0.045 |
| *t* | -0.366 | -1.089 | -0.414 | -0.877 | -0.649 | -0.557 | -0.698 | -0.816 |
| *P* | 0.36 | 0.14 | 0.34 | 0.19 | 0.26 | 0.29 | 0.24 | 0.21 |

Significance values are one-tailed (df = 30). L, R: left, right.
